# Retinal Microvascular Health: Can the Eye Tell Us About the Brain?

**DOI:** 10.1093/geroni/igaa057.2923

**Published:** 2020-12-16

**Authors:** Alison Abraham, Xinxing Guo, Xiangrong Kong, A Richey Sharrett, Brandon Lujan, David Huang, Pradeep Ramulu

**Affiliations:** 1 Johns Hopkins University School of Medicine, Baltimore, Maryland, United States; 2 Johns Hopkins University, Baltimore, Maryland, United States; 3 Casey Eye Institute, Oregon Health and Science University, Portland, Oregon, United States; 4 Johns Hopkins School of Medicine, Baltimore, Maryland, United States

## Abstract

Cognitive impairment caused by Alzheimer’s and other dementias is linked to vascular damage in the brain. Early microvascular changes in the brain are difficult to detect but in the retina, they can potentially be seen using optical coherence tomographic angiography. In cross-sectional analysis, associations between retinal vessel density (VD) and cognitive performance were assessed by regression analysis of cognitive function z-scores (for global function as well as language, memory and executive function domains) on VD, controlling for age, race and education. Among 177 participants without dementia (50% black; 67% female; mean age 78 years [range: 71-93 years]), the mean (SD) superficial vascular complex VD was 48.0% (7.2). Among the 191 eyes without eye disease, there were no significant associations of VD with global or domain specific cognitive function. Early changes in the eye related to systemic disease processes may not currently be detectable in healthy older adults with imaging data.

